# Improving patient safety culture in Saudi Arabia (2012–2015): trending, improvement and benchmarking

**DOI:** 10.1186/s12913-017-2461-3

**Published:** 2017-08-02

**Authors:** Khalid Alswat, Rawia Ahmad Mustafa Abdalla, Maher Abdelraheim Titi, Maram Bakash, Faiza Mehmood, Beena Zubairi, Diana Jamal, Fadi El-Jardali

**Affiliations:** 1 King Khalid University Hospital, King Saud University Medical City, Riyadh, Saudi Arabia; 2 Department of Health Management and Policy, American University of Beirut, Beirut, Lebanon; 3 Department of Health Research Methods, Evidence, and Impact, McMaster University, CRL-209, 1280 Main St. West, Hamilton, ON L8S 4K1 Canada

**Keywords:** Patient safety culture, Riyadh, Trending, Benchmarking

## Abstract

**Background:**

Measuring patient safety culture can provide insight into areas for improvement and help monitor changes over time. This study details the findings of a re-assessment of patient safety culture in a multi-site Medical City in Riyadh, Kingdom of Saudi Arabia (KSA). Results were compared to an earlier assessment conducted in 2012 and benchmarked with regional and international studies. Such assessments can provide hospital leadership with insight on how their hospital is performing on patient safety culture composites as a result of quality improvement plans. This paper also explored the association between patient safety culture predictors and patient safety grade, perception of patient safety, frequency of events reported and number of events reported.

**Methods:**

We utilized a customized version of the patient safety culture survey developed by the Agency for Healthcare Research and Quality. The Medical City is a tertiary care teaching facility composed of two sites (total capacity of 904 beds). Data was analyzed using SPSS 24 at a significance level of 0.05. A t-Test was used to compare results from the 2012 survey to that conducted in 2015. Two adopted Generalized Estimating Equations in addition to two linear models were used to assess the association between composites and patient safety culture outcomes. Results were also benchmarked against similar initiatives in Lebanon, Palestine and USA.

**Results:**

Areas of strength in 2015 included Teamwork within units, and Organizational Learning—Continuous Improvement; areas requiring improvement included Non-Punitive Response to Error, and Staffing. Comparing results to the 2012 survey revealed improvement on some areas but non-punitive response to error and Staffing remained the lowest scoring composites in 2015. Regression highlighted significant association between managerial support, organizational learning and feedback and improved survey outcomes. Comparison to international benchmarks revealed that the hospital is performing at or better than benchmark on several composites.

**Conclusion:**

The Medical City has made significant progress on several of the patient safety culture composites despite still having areas requiring additional improvement. Patient safety culture outcomes are evidently linked to better performance on specific composites. While results are comparable with regional and international benchmarks, findings confirm that regular assessment can allow hospitals to better understand and visualize changes in their performance and identify additional areas for improvement.

## Background

Patient safety (PS) and the prevention of harm has been linked to developing a strong patient safety culture (PSC) [1]. Creating and maintaining a strong PSC in healthcare organizations is linked to better performing health organizations [2].

Evidence on patient safety culture in hospitals can provide healthcare leaders and policymakers with the information they need to improve quality and prevent errors. Administrators, managers and policymakers alike will reap the benefits of improving patient safety culture in improved quality, improved patient outcomes, reduced errors and a more cost effect healthcare system [1, 3–6].

Patient safety culture is determined by multiple factors within a health organization and can support the prevention and reduction of harms to patients. It is the outcome of different factors within a healthcare institution including attitudes, values, skills and even behaviors to commit to patient safety management [7].

International accreditation organizations are now requiring PSC assessments as an integral component of their surveys and provide important information that would help better understand overall organizational perception on areas related to PS [8, 9]. So in response to these requirements, many hospitals around the world are using different tools for redesigning and restructuring their work environments to support safe job performance and promote PSC [9]. The *Hospital Survey on Patient Safety Culture* (HSOPSC) has become the most frequently used tool to assess patient safety culture [10]. This tool measures different aspects of patient safety culture and can help hospitals better understand the factors that determine how they relate to their actions, managerial support, organizational activities, feedback about errors, communication, teamwork within and across units, staffing, handoffs and response to error [10]. In spite of the abundance of literature and evidence that attests to the importance of patient safety culture assessments, this topic has not been sufficiently addressed in in the Arab world and particularly in the Kingdom of Saudi Arabia (KSA). The existing evidence about KSA found that organizational learning [11, 12], teamwork within units, in addition to feedback and communication about errors are among the strongest aspects of patient safety culture [12]. On the other hand, and in accordance with international trends, punitive response to error [11, 12] staffing, and teamwork across units are some of the areas requiring improvement [12]. Evidence from a multi-site facility in Riyadh also confirmed that the composites on organizational learning, and teamwork within units were areas of strength while punitive response to error, staffing and communication were areas of weakness [13].

In Lebanon, a national study that targeted hospital employees used an adapted Arabic version of the HSOPSC. The study found that teamwork within units, hospital management support for patient safety, and organizational learning and continuous improvement were areas of strength. Areas requiring improvement at the national level were teamwork across hospital units, hospital handoffs and transitions, staffing, and non-punitive response to error [14]. The study also found significant associations between patient safety culture outcomes and composite scores [15].

A study in Oman focusing on patient safety culture from the nursing perspective found perception of patient safety was associated with better scores on supervisor or manager expectations, feedback and communications about errors, teamwork across hospital units, and hospital handoffs and transitions [4]. Another study focusing on public hospitals in Palestine found that the composites with the lowest scores were non-punitive response to error, frequency of events reported, communication openness, hospital management support for patient safety and staffing [16].

Assessments of patient safety culture using the Agency for Healthcare Research and Quality (AHRQ) tool should ideally be repeated every two or 3 years [17, 18]. This recommendation was also highlighted in the Saudi Central Board for Accreditation of Healthcare Institutions (CBAHI) accreditation standards which recommends conducting a patient safety culture assessment on an annual basis [19]. We have yet to document a study that has conducted and reported such repeated assessments in the Eastern Mediterranean Region, and specifically in the Kingdom of Saudi Arabia. Such assessments can provide hospital management and higher leadership with some insight on how their performance has changed as a result of quality improvement plans that were developed in response to the findings of the patient safety culture survey.

This particular study is a second round assessment of a previous patient safety culture survey conducted in 2012. This study focused on the same multi-site facility in an effort to determine whether performance on patient safety culture composites has changed. The current study also compares results to the previous assessment in 2012 in addition to benchmarking to other initiatives conducted regionally and internationally. To our knowledge, this is the first study to perform this type of assessment in the context of Kingdom of Saudi Arabia (KSA) and Arab countries.

### Objectives

We aim to re-assess PSC in a large multi-site healthcare facility in Riyadh, Kingdom of Saudi Arabia and to compare it with an earlier assessment conducted in 2012 and benchmarked against regional and international studies. Furthermore, we explored the association between PSC predictors and outcomes while considering demographic characteristics and hospital size.

## Methods

### Design, setting and sampling

The tool used was adapted from the Hospital Survey on Patient Safety Culture (HSOPSC) developed by the Agency for Healthcare Research and Quality. The survey is available in English and was translated to Arabic in a previous study conducted in Lebanon [14]. The research team piloted the translated version in 2012 survey and made minor changes to the wording of some statements to better fit the context of the hospital. The changes were cross checked with the English version to make sure not to alter the original meaning [13]. Minimal changes were made to the current version and they only related to categories of employment.

The Medical City is a tertiary care teaching hospital with a capacity of 800 beds. It has a wide range of specialties and services and serves patients from all over KSA. The facility is divided into two settings: the larger setting (Site A) has 700 beds and the smaller setting (Site B) has 100 beds. Site A is located towards the North of Riyadh and offers free medical services with a wide range of specialties. Site B is located towards the center of Riyadh and was the first educational hospital in Saudi Arabia but offers fewer services compared to Site A given its smaller size. The Dental Site is within Site A and offers inpatient and outpatient dental services.

The survey randomly sampled staff including physicians, registered nurses, other clinical or non-clinical staff, pharmacists, laboratory technicians, dietary department staff, radiologists, and administrative staff including managers and supervisors. The two sites had a total of 9000 hospital employees of which 4500 were targeted and 2592 responded to the survey (response rate of 57.6%). Data collection spanned July 2015 to December 2015. The survey was available in electronic format for all respondents. Some respondents preferred paper based surveys and as such were provided with the surveys in sealed envelopes. A total of 397 respondents returned the completed surveys in designated boxes in sealed envelopes to maintain the confidentiality of their responses. The consent form was included on the first page of the survey and detailed the information for participants and some definitions. Respondents were asked not to write their names or sign any section of survey.

Surveys were provided in both English and Arabic with respondents favoring the English version. Data was not collected on language for either the online version of the survey. It should be noted, however, that the ratio of English to Arabic surveys in the paper based version was 3 to 1 which confirms preference of the English version.

### Data management and analysis

Data was analyzed using IBM SPSS Statistics 24.0 at a significance level of 0.05. The tool included a total of 44 items, 42 of them measure 12 patient safety culture composites (two of which are patient safety culture outcomes). The tool includes four outcomes, two of which are included within the composites, they are: frequency of events reported and overall perception of patient safety. The two other outcome variables are patient safety grade and number of events both of which are multiple choice questions. The HSOPSC includes both positively and negatively worded items scored using a five-point scale reflecting agreement or frequency of occurrence on a five-point Likert scale. The total percent positives, negatives and neutrals were calculated for each composite making sure to reverse negatively worded items [18]. Composites that had at least 70% positive response was considered an area of strength whereas those scoring less were considered areas for improvement.

Composite level scores were also calculated. This was done through adding up the score for each item within a composite then dividing by the number of non-missing items within the scale. Computed scores ranged from 1 to 5. Internal consistency was measured using Cronbach’s alpha.

Confirmatory Factor Analysis was conducted results confirmed that 9 of the 12 composites loaded on one factor with acceptable eigen values and percent variance explained. The three composites supervisor/manager expectations, overall perception of patient safety, and staffing each loaded on two factors. Detailed results are not reported in this paper.

Demographic characteristics of respondents were summarized using univariate analysis.

In fulfillment of the comparative component within this study, the two datasets were merged to combine survey items from the 2012 survey with those of the 2015 survey. Only scale related items were merged, demographics were not included. A Student T-Test was used to examine whether a statistically significant different exists between the survey items for each of 2012 and 2015 datasets.

Results from this hospital were also benchmarked against similar initiatives in the United States (US) [17] and Lebanon [15]. Comparison to the benchmark value was done using the below formula [20]:

$$ \%\mathrm{Distance}\ \mathrm{from}\ \mathrm{benchmark}={\left(\left(\mathrm{benchmark}\ \mathrm{value}\hbox{--} \mathrm{hospital}\ \mathrm{result}\right)/\mathrm{benchmark}\ \mathrm{value}\right)}^{\ast }\ 100. $$

Categories of achievement were determined by the value of % distance from benchmark as follows:

➢ Values <10% were categorized as **Meets or better than benchmark (☑).** Values below zero (0) indicate that the benchmark value is lower than the hospital result thus giving a result of “meet or better than benchmark”.

➢ Values between [10–50%] were categorized as **Deviates slightly from benchmark (▣).**

➢ Values exceeding 50% were categorized as **Major deviation from benchmark (☒).**

The four outcome variables were regressed against the 10 composite scores, respondent’s gender, age, experience, degree, respondent position, patient interaction and size of the hospital. Four regression models were used to analyze the association between the composites and the outcome variables. The first two models were Generalized Estimating Equations which included recoded versions of the variables on number of events reported and patient safety grade. These two outcomes were reduced to include three items each. Patient safety grade was reduced to include the categories: “Poor or Failing,” “Acceptable,” and “Excellent/Good.” Number of events was reduced to include: “>5 events reported,” “1 to 5 events reported,” and “No events reported.” Linear regression was used for the two composites on frequency of events and overall perception of patient safety. For the purpose of linear regression, the independent variables were entered as dummy variables.

## Results

### General results

A total of 4500 surveys were sent to respondents of which 2592 completed (2128 from Site A and 441 from Site B, in addition to a total of 23 respondents from dental and combined sites) yielding an overall response rate of 56.7%.

Analysis revealed that the majority of respondents were females (84.1%) and around half were aged between 30 and 45 (46.4%) and married (64.4%). Around half the respondents indicated working in Medical departments (51.9%) while 30.6% worked in Surgical departments. The majority of respondents indicated working as Registered Nurses (78.3%) (Table 1). Most respondents reported holding a Bachelor’s degree (56.2%) and having 3 to 5 years of experience (25.2%) at the hospital, 6 to 10 years of experience in their work area (31.5%) and 6 to 10 years of experience in their profession (32.3%). Most respondents indicated working 40 to 60 h a week (92.9%) and having direct contact with patients (90.9%).

**Table 1:** Socio-demographic and professional characteristics of respondents in addition to frequency of events and patient safety grade

	N (%)
Gender	
Male	398 (15.9%)
Female	2103 (84.1%)
Age group	
Below 30 year old	925 (37.3%)
Between 30 to 45 years old	1152 (46.4%)
Between 46 to 55 years old	253 (10.2%)
Above 55 years old	151 (6.1%)
Marital Status	
Single	851 (34.2%)
Married	1602 (64.4%)
Divorced/ Separated	16 (0.6%)
Widowed	13 (0.5%)
Others	6 (0.2%)
Highest Education	
Under High School Level	2 (0.1%)
High School Level	7 (0.3%)
Diploma Level	836 (33.5%)
Bachelor’s Degree	1403 (56.2%)
Master’s Degree	127 (5.1%)
Doctorate Degree	102 (4.1%)
Others	19 (0.8%)
Work Area	
Many different hospital unit/No Specific Unit	21 (0.8%)
Administrative	138 (5.4%)
Medical	1332 (51.9%)
Surgical	786 (30.6%)
Diagnostics	99 (3.9%)
Other	191 (7.4%)
Staff Position	
Administrator/Manager/Director	47 (1.9%)
Physician	141 (5.6%)
Specialist	61 (2.4%)
Coordinator	10 (0.4%)
Assistant/Aide	39 (1.6%)
Pharmacist	36 (1.4%)
Therapist	1 (0%)
Registered Nurse	1969 (78.3%)
Resident/PG/Intern	64 (2.5%)
Assistant/Clerk/Secretary/Facilitator	28 (1.1%)
Technician	52 (2.1%)
Other, please specify:	67 (2.7%)
Tenure in Profession	
Less than 1 year	133 (5.3%)
1 to 5 years	741 (29.6%)
6 to 10 years	809 (32.3%)
11 to 15 years	348 (13.9%)
16 to 20 years	222 (8.9%)
21 years or more	252 (10.1%)
Hours worked per week	
Less than 20 h per week	25 (1%)
20 to 39 h per week	148 (6%)
40 to 60 h per week	2280 (92.9%)
Contact with Patients	
YES, I typically have direct interaction or contact with patients.	2229 (90.9%)
NO, I typically do NOT have direct interaction or contact with patients.	224 (9.1%)
Patient Safety Grade	
A – Excellent	495 (19.3%)
B - Very Good	1235 (48.1%)
C – Acceptable	650 (25.3%)
D – Poor	51 (2.0%)
E – Failing	5 (0.2%)
Missing	133 (5.2%)
Frequency of Events	
No event reports	1352 (55.8%)
1 to 2 event reports	678 (28.0%)
3 to 5 event reports	257 (10.6%)
6 to 10 event reports	76 (3.1%)
11 to 20 event reports	30 (1.2%)
21 event reports or more	32 (1.3%)
Missing	144 (5.9%)

Less than half the respondents gave their hospital a Very Good patient safety grade (49.4%) while 55.8% reported no events (55.8%), 27.8% reported 1 to 2 events, and 10.6% reported 3 to 5 events. It is worth noting that only 1.3% of respondents reported 21 or more events (Table 1).

### Areas of strengths and areas requiring improvement

Areas of strength (those where percent positive rating exceeds 70%) and those requiring improvement (scoring below 70%) were then examined [10]. The dimensions considered areas of strength were Teamwork within units (84.8%), Organizational Learning – Continuous Improvement (86.3%), Management support for patient safety (75.3%) and Feedback and Communication about error (71.8%) (Table 2).

**Table 2:** Cronbach’s alpha and distribution of positive responses and scores for survey composites and items

Composites and survey items	Average% positive response^a^	Mean (Standard deviation)
Overall Perception of Safety (Cronbach’s α = 0.234)	59.5	3.41 (0.54)
It is just by chance that more serious mistakes do not happen around here (R)^b^	29.4	2.72 (1.06)
Patient safety is never sacrificed to get more work done	76.6	3.80 (0.97)
We have patient safety problems in this unit (R)	49.7	3.19 (1.09)
Our policies and procedures and systems are effective in preventing errors	82.1	3.91 (0.75)
Supervisor/Manager Expectations & Actions Promoting Patient Safety (Cronbach’s α = 0.395)	60.8	3.44 (0.60)
My supervisor/manager says a good word when he/she sees a job done according to established patient safety procedures	74.2	3.74 (0.94)
My supervisor/manager seriously considers staff suggestions for improving patient safety	76.4	3.80 (0.87)
Whenever pressure builds up, my supervisor/manager wants us to work faster, even if it means taking shortcuts (R)	52.1	3.27 (1.06)
My supervisor/manager overlooks patient safety problems that happen over and over (R)	40.4	2.94 (1.16)
Organizational learning and Continuous Improvement (Cronbach’s α = 0.614)	86.3	4.03 (0.53)
We are actively doing things to improve patient safety	94.8	4.31 (0.64)
Mistake have led to positive changes here	76.8	3.78 (0.78)
After we make changes to improve patient safety, we evaluate their effectiveness	87.4	4.01 (0.69)
Teamwork within units (Cronbach’s α = 0.757)	84.8	3.40 (0.60)
Staff support one another in this unit	90.1	4.11 (0.71)
When a lot of work needs to be done quickly, we work together as a team to get the work done	89.3	4.11 (0.71)
In this unit, people treat each other with respect	85.4	4.03 (0.75)
When members of this unit get really busy, other members of the same unit help out	74.2	3.75 (0.95)
Non-punitive Response to Error (Cronbach’s α = 0.694)	24.8	2.62 (0.79)
Staff feel like their mistakes are held against them (R)	31.4	2.82 (1.04)
When an event is reported, it feels like the person is being written up, not the problem (R)	29.3	2.76 (1.02)
Staff worry that mistakes they make are kept in their personnel file (R)	13.7	2.29 (0.93)
Staffing (Cronbach’s α = 0.210)	33.8	2.79 (0.57)
We have enough staff to handle the workload	56.1	3.29 (1.18)
Staff in this unit work longer hours than is best for patient care (R)	11.2	2.17 (0.89)
We use agency/temporary staff than is best for patient care (R)	45.2	3.14 (1.08)
When the work is in “crisis mode” we try to do too much, too quickly (R)	22.8	2.56 (1.01)
Hospital Management Support for Patient Safety (Cronbach’s α = 0.519)	75.3	3.76 (0.62)
Hospital management provides a work climate that promotes patient safety	85.3	3.95 (0.68)
The actions of hospital management show that patient safety is a top priority	86.9	4.07 (0.77)
Hospital management seems interested in patient safety only after an adverse event happens (R)	53.6	3.26 (1.10)
Teamwork Across Hospital Units (Cronbach’s α = 0.627)	67.0	3.59 (0.62)
There is good cooperation among hospital units that need to work together	73.0	3.69 (0.82)
Hospital units work well together to provide the best care for patients	85.5	4.03 (0.77)
Hospital units do not coordinate well with each other and this might affect patient care (R)	55.8	3.30 (1.04)
It is often not easy to work with staff from other hospital units (R)	53.7	3.35 (0.97)
Hospital Handoffs & Transitions (Cronbach’s α = 0.783)	55.8	3.39 (0.75)
Things “fall between the cracks”, i.e., things might go uncontrolled and get lost (ex: medical records, medical treatment, patient information and education, discharge criteria) when transferring patients from one unit to another (R)	45.5	3.18 (1.01)
Important patient care information is often lost during shift changes (R)	66.8	3.59 (0.96)
Problems often occur in the exchange of information across hospital units (R)	46.2	3.22 (0.95)
Shift changes are problematic for patients in this hospital (R)	64.5	3.56 (0.93)
Communication Openness (Cronbach’s α = 0.533)	45.0	3.36 (0.83)
Staff will freely speak up if they see something that may negatively affect patient care	64.5	3.84 (1.07)
Staff feel free to question the decisions or actions of those with more authority	34.3	3.08 (1.21)
Staff are afraid to ask questions when something does not feel right (R)	36.2	3.15 (1.15)
Feedback and Communications About Error (Cronbach’s α = 0.732)	71.8	4.04 (0.79)
We are given feedback about changes put into place based on event reports	56.5	3.69 (1.06)
We are informed about errors that happen in this unit	79.0	4.21 (0.95)
In this unit, we discuss ways to prevent errors from happening again	79.9	4.22 (0.93)
Frequency of events reported (Cronbach’s α = 0.902)	68.8	3.92 (1.10)
When a mistake is made, but is caught and corrected affecting the patient, how often is this reported?	65.6	3.83 (1.21)
When a mistake is made, but has no potential to harm the patient, how often is this reported?	65.9	3.86 (1.22)
When a mistake is made that could harm the patient, but does not, how often is this reported?	74.9	4.07 (1.17)

^a^the composite-level percentage of positive responses was calculated using the following formula: (number of positive responses to the items in the composite/total number of responses to the items (positive, neutral, and negative) in the composite (excluding missing responses))*100

^b^Negatively worded items that were reverse coded

Areas of strength and those requiring improvement were derived. A major area of strength highlighted in the survey findings included the degree to which the hospital is engaging in actions to improve patient safety (94.8% positive). Additional areas of strength were revealed within the composite on Teamwork within units. Respondents indicated that staff support each other within the unit (90.1% positive responses), and work together as a team (89.3% percent positive). Moreover, as highlighted within the composite on Hospital Management Support for Patient Safety, 86.9% of respondents indicated that the actions of hospital management reflect that patient safety is a priority for the administration (Table 2).

Areas requiring improvement related to staffing. In fact, respondents indicated that hospital employees work longer than what should be considered best for patient safety (11.2% positive response). As for the dimension on Non-Punitive Response to Error, 13.7% of staff were worried that their mistakes were being kept in their personnel file and 29.3% felt that they were being written up when reporting an event (Table 2). Other items that reflect areas of strength and items requiring improvement are listed in Table 2.

### Comparing results from 2015 to 2012

The difference in mean scores on the survey composites was statistically significant between 2012 and 2015. Results improved on all survey composites indicating better performance in 2015 compared to the initial survey. Non-punitive response to error and Staffing remained the lowest scoring composites in 2015. The highest ranking composite for both surveys were Organizational Learning-Continuous Improvement. While Teamwork within Units had the second highest score in 2012, it ranked third in 2015 while Feedback and Communication about Errors ranked second (Table 3).

**Table 3:** T-test to compare composite scores in 2012 to scores in 2015

	2012		2015		*P*-value
	Mean	SD	Mean	SD	
Frequency of Event Reporting	3.64	1.16	4.04	1.54	<0.001
Overall Perceptions of Safety	3.43	0.59	3.60	1.56	<0.001
Supervisor/manager expectations and actions promoting safety	3.46	0.65	3.57	1.34	<0.001
Organizational Learning-Continuous Improvement	3.89	0.69	4.16	1.14	<0.001
Teamwork Within Hospital Units	3.85	0.75	4.04	0.71	<0.001
Communication Openness	3.25	0.85	3.45	1.08	<0.001
Feedback and Communication About Errors	3.73	0.95	4.11	1.10	<0.001
Non-punitive Response to Error	2.68	0.81	2.76	1.26	0.013
Staffing	2.84	0.62	3.02	1.19	<0.001
Hospital Management Support for Patient Safety	3.69	0.76	3.85	1.05	<0.001
Hospital Handoffs and Transitions	3.36	0.79	3.82	2.29	<0.001
Teamwork Across Hospital Units	3.52	0.71	3.76	1.36	<0.001

### Comparative against regional and international findings

Composite scores were compared to similar studies done in Lebanon, Palestine and United States. As compared to the US, the Medical City in Riyadh was found to meet or exceed benchmarks for dimension pertaining to Teamwork within Units, Organizational Learning—Continuous Improvement, Management Support for Patient Safety, Feedback and Communication About Error, Frequency of Events Reported Staffing, and Non-Punitive Response to Error (Table 4).

**Table 4:**
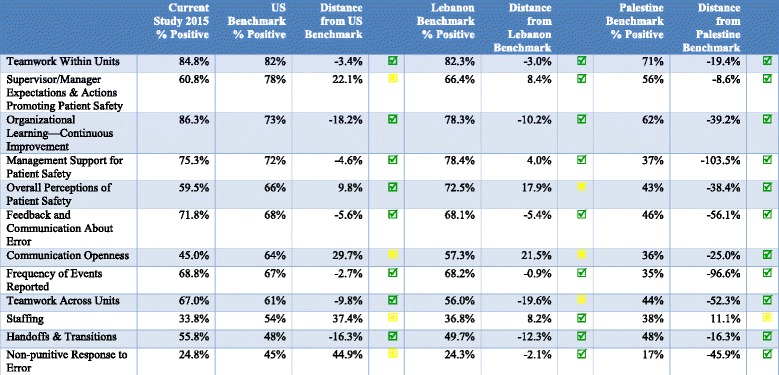
Benchmarking 2015 results to similar initiatives in the US and Lebanon

Compared to Lebanon, the Medical City in Riyadh fared better on dimensions relating to Teamwork Within Units, Teamwork across units, Supervisor/Manager Expectations & Actions Promoting Patient Safety, Organizational Learning—Continuous Improvement, Management Support for Patient Safety, Feedback and Communication about Error, Frequency of Events Reported, Staffing, Handoffs & Transitions and Non-punitive Response to Error (Table 4).

Results from the Medical City were found to be better than the Palestine benchmark with the exception of the composite relating to Staffing (Table 4).

#### Generalized estimating equations findings patient safety grade

Table 5 shows how increases in patient safety composite scores affect outcomes. A one unit increase on all patient safety composites with the exception of non-punitive response to error significantly increased odds of reporting better patient safety grades. A one-unit increase in staffing had 1.04 higher odds of reporting better patient safety grade (95% CI = 1.01–1.08). A one unit increase on remaining composites increased odds of reporting better patient safety grade ranging from an OR of 1.12 to 1.66. Noteworthy is the finding that a one unit increase on Hospital Management Support for Patient Safety had 2.43 higher odds of reporting better patient safety grade (95% CI = 2.09–2.83) (See Table 5).

**Table 5:** Generalized estimating equations

	Patient safety grade		Number of events reported	
	OR (95% CI)	*P*-value	OR (95% CI)	*P*-value
Patient Safety Culture Composites				
Supervisor/Manager Expectations & Actions Promoting Patient Safety	1.20 (1.19–1.22)	<0.001	1.27 (0.91–1.78)	0.162
Organizational learning and Continuous Improvement	1.66 (1.55–1.77)	<0.001	1.10 (0.99–1.22)	0.073
Teamwork within units	1.61 (1.59–1.62)	<0.001	0.82 (0.76–0.89)	<0.001
Communication Openness	1.22 (1.10–1.35)	<0.001	0.93 (0.90–0.97)	0.002
Feedback and Communications About Error	1.50 (1.29–1.74)	<0.001	0.99 (0.97–1.01)	0.282
Non-punitive Response to Error	1.09 (0.93–1.27)	0.308	0.83 (0.71–0.98)	0.029
Staffing	1.04 (1.01–1.08)	0.007	0.74 (0.67–0.83)	<0.001
Hospital Management Support for Patient Safety	2.43 (2.09–2.83)	<0.001	1.15 (1.08–1.23)	<0.001
Hospital Handoffs & Transitions	1.12 (1.11–1.13)	<0.001	1.10 (1.06–1.14)	<0.001
Teamwork Across Hospital Units	1.48 (1.45–1.50)	<0.001	0.94 (0.90–0.98)	0.004
Gender				
Male	1		1	
Female	0.62 (0.62–0.63)	<0.001	1.56 (1.45–1.67)	<0.001
Age				
Less than 30 years of age	1		1	
Between 30 and 45	1.06 (0.92–1.23)	0.423	0.96 (0.81–1.14)	0.641
Between 46 and 55	1.00 (0.72–1.40)	0.995	0.56 (0.46–0.69)	<0.001
Aged above 55	1.28 (1.14–1.42)	<0.001	0.40 (0.38–0.43)	<0.001
Experience at the hospital				
1 to 2 years	1		1	
3 to 5 years	0.96 (0.85–1.08)	<0.001	1.54 (1.21–1.94)	<0.001
6 to 10 years	0.67 (0.57–0.78)	0.291	1.44 (1.35–1.53)	<0.001
11 to 15 years	1.07 (1.01–1.14)	0.025	1.55 (1.51–1.59)	<0.001
16 to 20 years	0.78 (0.49–1.24)	<0.001	2.38 (1.54–3.68)	<0.001
More or equal to 21 years	1.58 (1.55–1.60)	0.463	3.34 (2.53–4.41)	<0.001
Highest Degree				
Under High School Level	-	-	1	
High school level	-	-	0.50 (0.19–1.28)	0.148
Diploma level	-	-	0.36 (0.12–1.09)	0.070
Bachelors Degree	-	-	0.65 (0.26–1.62)	0.354
Masters Degree	-	-	0.49 (0.30–0.81)	0.005
Doctorate Degree	-	-	0.33 (0.17–0.65)	0.001
Position at the hospital				
Administrator/Manager/Director	1		1	
Physician	0.50 (0.40–0.64)	<0.001	0.43 (0.35–0.51)	<0.001
Specialist	1.65 (0.74–3.67)	0.223	0.32 (0.24–0.43)	<0.001
Coordinator	0.65 (0.65–0.65)	<0.001	1.01 (0.58–1.76)	0.964
Assistant/Aide	1.89 (1.34–2.67)	<0.001	0.27 (0.23–0.31)	<0.001
Pharmacist	0.53 (0.52–0.55)	<0.001	2.97 (2.30–3.84)	<0.001
Registered Nurse	0.60 (0.57–0.64)	<0.001	0.29 (0.17–0.50)	<0.001
Resident/PG/Intern	0.18 (0.14–0.22)	<0.001	0.14 (0.11–0.18)	<0.001
Assistant/Clerk/Secretary/Facilitator	0.65 (0.65–0.65)	0.611	1.02 (0.91–1.14)	0.734
Technician	2.84 (2.49–3.24)	<0.001	0.55 (0.53–0.58)	<0.001
Other	1.04 (0.98–1.12)	0.189	0.19 (0.06–0.63)	0.006
Interaction with patients				
No	0.81 (0.74–0.88)	<0.001	0.64 (0.54–0.76)	<0.001
Yes	1		1	
Hospital Size				
Small	0.56 (0.56–0.57)	<0.001	0.87 (0.87–0.87)	<0.001
Large	1		1	

Female respondents had 0.62 lower odds (95% CI = 0.62–0.63) of reporting better patient safety grades while those aged above 55 had 1.28 higher odds of reporting better patient safety grades (95% CI = 1.14–1.42). Work experience was associated with higher patient safety grades whereby 3 to 5 years of experience was associated with 0.96 lower odds of reporting better patient safety grades whereas respondents with 11 to 15 years or 16 to 20 years of experience had significantly greater odds of reporting better patient safety grades (See Table 5). Respondent positions such Physicians, Coordinators, Pharmacist, Nurses, and Resident/PG/Intern were all associated with lower odds of reporting better patient safety grades. However, Assistant/Aide and Technicians had higher odds of reporting better patient safety grades. Respondents who did not have patient interaction and those working in the smaller setting also had lower odds of reporting better patient safety grades (See Table 5).

#### Number of events reported

A one unit increase in Hospital Management Support for Patient Safety had 1.15 higher odds of reporting higher number of events (95% CI = 1.08–1.23). Moreover, a one unit increase in Hospital Handoffs and Transitions had 1.10 higher odds of reporting higher number of events (95% CI = 1.06–1.14). Teamwork within units, Communication Openness, Non-punitive Response to Error, Staffing, and Teamwork across Hospital Units were all associated with lower odds of reporting higher number of events (See Table 5).

Female respondents had 1.56 higher odds (95%CI = 1.45–1.67) of reporting higher number of events. Respondents aged 46 and above were found to have significantly lower odds of reporting higher number of events. This observation is reversed when it comes to years of experience where more experienced respondents had consistently higher odds of reporting higher number of events. Moreover, respondents holding Masters or Doctoral degrees had significantly lower odds of reporting higher number of events. As for respondent positions, Physicians, Specialists, Assistant/Aide, Registered Nurse, Resident/PG/Intern, Technicians and Other all had significantly lower odds of reporting higher number of events. However, Pharmacists had 2.97 higher odds of reporting higher number of events (95% CI = 2.30–3.84). As expected, respondents who had no patient interaction had 0.64 lower odds of reporting higher number of events. The smaller hospital also had significantly lower odds of reporting higher number of events (OR = 0.87, 95% CI = 0.87–0.87) (See Table 5).

### Linear regression findings

#### Overall perception of safety

Perception of patient safety improved by 0.131 (*P*-Value <0.001) for a one unit increase in the score on Supervisor/Manager Expectations and Actions Promoting Safety, by 0.10 (*P*-Value =0.003) for every unit increase in the score on organizational learning and continuous improvement, and by 0.052 (*P*-Value =0.007) for a one unit increase in the score on Non-Punitive Response to Error. A one unit increase in the composites on Staffing, Hospital Management Support for Patient Safety, Hospital Handoffs & Transitions were also found to increase overall perception of patient safety by 0.079 (*p*-value =0.002, 0.114 (*p*-value <0.001) and 0.12 (*p*-value <0.001) (See Table 6).

**Table 6:** Linear regression model

	Perception of patient safety		Frequency of events reported	
	Beta (Standard error)	*P*-value	Beta (Standard error)	*P*-value
Patient Safety Culture Composites				
Supervisor/ Manager Expectations & Actions Promoting Patient Safety	0.131 (0.027)	<0.001	−0.009 (0.057)	0.880
Organizational learning and Continuous Improvement	0.100 (0.034)	0.003	0.133 (0.071)	0.060
Teamwork within units	0.055 (0.029)	0.059	−0.107 (0.061)	0.080
Communication Openness	−0.026 (0.020)	0.181	−0.004 (0.041)	0.922
Feedback and Communications About Error	0.008 (0.022)	0.728	0.431 (0.046)	<0.001
Non-punitive Response to Error	0.052 (0.019)	0.007	−0.061 (0.04)	0.125
Staffing	0.079 (0.026)	0.002	−0.017 (0.054)	0.748
Hospital Management Support for Patient Safety	0.114 (0.030)	<0.001	0.119 (0.063)	0.056
Hospital Handoffs & Transitions	0.120 (0.023)	<0.001	−0.031 (0.047)	0.515
Teamwork Across Hospital Units	0.003 (0.032)	0.926	−0.014 (0.066)	0.834
Gender				
Male	0.142 (0.054)	0.008	0.048 (0.11)	0.667
Female	0		0	
Age				
Less than 30 years of age	0		0	
Between 30 and 45	−0.055 (0.035)	0.122	−0.172 (0.074)	0.021
Between 46 and 55	−0.137 (0.068)	0.042	0.022 (0.143)	0.880
Aged above 55	−0.203 (0.094)	0.031	0.16 (0.195)	0.412
Experience at the hospital				
1 to 2 years	0		0	
3 to 5 years	0.038 (0.036)	0.287	0.136 (0.075)	0.071
6 to 10 years	0.010 (0.045)	0.825	0.202 (0.094)	0.031
11 to 15 years	0.061 (0.056)	0.278	0.173 (0.117)	0.140
16 to 20 years	0.046 (0.089)	0.607	0.03 (0.185)	0.871
More or equal to 21 years	0.086 (0.091)	0.345	0.048 (0.19)	0.801
Highest Degree				
Under High School Level	0		0	
High school level	0.483 (0.237)	0.042	−0.288 (0.493)	0.559
Diploma level	0.528 (0.237)	0.026	−0.326 (0.492)	0.508
Bachelors Degree	0.583 (0.247)	0.019	0.076 (0.513)	0.882
Masters Degree	0.569 (0.258)	0.027	−0.231 (0.533)	0.665
Doctorate Degree	0.271 (0.283)	0.339	−0.105 (0.597)	0.861
Position at the hospital				
Administrator/Manager/Director	0.229 (0.123)	0.064	−0.656 (0.269)	0.015
Physician	−0.167 (0.099)	0.093	−0.597 (0.203)	0.003
Specialist	−0.292 (0.128)	0.023	−0.858 (0.265)	0.001
Coordinator	0.152 (0.216)	0.482	0.356 (0.449)	0.429
Assistant/Aide	0.001 (0.101)	0.995	0.037 (0.21)	0.860
Pharmacist	0.083 (0.152)	0.585	−0.37 (0.332)	0.266
Registered Nurse	−0.161 (0.109)	0.143	−0.499 (0.218)	0.023
Resident/PG/Intern	0.084 (0.181)	0.641	−0.279 (0.404)	0.490
Assistant/Clerk/Secretary/Facilitator	0.006 (0.121)	0.961	−0.478 (0.24)	0.046
Technician	−0.052 (0.100)	0.607	−0.096 (0.213)	0.652
Other	0		0	
Interaction with patients				
No	0		0	
Yes	0.068 (0.062)	0.273	0.108 (0.13)	0.406
Hospital Size				
Small	0		0	
Large	−0.098 (0.034)	0.004	−0.087 (0.071)	0.223

As age of respondents increased, overall overall perception of patient safety progressively decreased. However, respondents with higher educational degrees had significantly better perception of patient safety. Specialists and respondents working in the larger site also had significantly lower overall perception of patient safety (Table 6).

#### Frequency of events reported

Linear regression analysis showed that a one unit increase in the score on Feedback and Communications about Error increased the frequency of events reported by 0.431 (*P*-Value <0.001) (See Table 6).

Respondents aged between 30 and 45 years reported −0.172 fewer events (*p*-value =0.021) compared to respondents aged below 30. Moreover, respondents with 6 to 10 years reported 0.202 more events (*p*-value = 0.031) compared to respondents with 1 to 2 years of experience. As for respondent positions, Administrator/Manager/Director, Physician, Specialist, Registered Nurses and Assistant/Clerk/Secretary/Facilitator were all significantly less likely to report higher number of events (See Table 6).

## Discussion

This is the first study to conduct a repeated assessment of patient safety culture in a country where a dearth of such studies exist. Findings confirm that tangible improvement has been achieved on some composites while other areas still require further work. These findings are of utmost importance in the context of KSA where such assessments are limited but can provide valuable information to hospital leaders on how performance has changed as a result of quality improvement plans. Study findings also provide recent data on patient safety culture in the context of a leading health provider in a major city in KSA.

When comparing study findings to previous studies, evidence indicated that Organizational Learning [11, 12], Teamwork within Units, and Feedback and Communication about Errors are among the strongest aspects of patient safety culture [12] whereas the highly Punitive Response to Error [11, 12] Staffing, and Teamwork across Hospital Units as areas requiring improvement [12]. Another study conducted at a multi-site facility in Riyadh confirmed Organizational Learning, and Teamwork within Units as areas of strength and Punitive Response to Error, Staffing and Communication as areas of weakness [13]. In Lebanon, Teamwork within Units, Hospital Management Support for Patient Safety, and Organizational Learning and Continuous Improvement were areas of strength. Areas requiring improvement at the national level were Teamwork across Hospital Units, Hospital Handoffs and Transitions, Staffing, and Non-punitive Response to Error [14]. The study also found significant associations between patient safety culture outcomes and composite scores [15]. A similar study in Oman found that higher Overall Perception of Patient safety was associated with better composite scores on Supervisor or Manager Expectations, Feedback and Communications about Errors, Teamwork across Hospital units, and Hospital Handoffs and Transitions [4]. In Jordan, the main area of strength was Teamwork within Units [21]. Another study focusing on public hospitals in Palestine found that the composites with the lowest scores were Non-punitive Response to Error, Frequency of Events Reported, Communication Openness, Hospital Management Support for Patient Safety and Staffing [16].

Results of this survey showcased areas of strength and those requiring improvement and also showed whether any changes can be observed compared to the previous assessment. Areas of strength in this assessment were Teamwork within units, Organizational Learning – Continuous Improvement, Management support for patient safety and Feedback and Communication about error; the last composite being a new addition compared to the previous assessment [13]. The findings on these composites in particular reflect commitment from hospital management to focus on feedback as a means of improving reporting. Moreover, the effect of size continues to impact survey outcomes with smaller hospitals showing better overall scores reflecting that the impact of fewer hierarchical and bureaucratic requirements serve to the benefit of the smaller setting [13].

It is worth noting that Non-punitive Response to Error remains the composite with the lowest score in 2015. This reflects a culture which places more emphasis on punishment in addressing errors; this reflects ineffective policies that cannot prevent errors, improve reporting and ultimately impact patient safety [22]. Studies show that fear of punishment would reduce frequency of error reporting among nurses [2] and this is confirmed in the regression results from this study.

Evidence links hospital cultures that foster sharing and reporting of errors to better patient safety and quality of care [23]. This should go hand in hand with addressing issues such as poor communication, lack of visible leadership, poor teamwork, lack of reporting systems, inadequate analysis of adverse events and inadequate staff knowledge about safety [4].

The study also benchmarked hospital performance to similar assessments in the US and Lebanon. While there are no major deviations from benchmarks, some areas of slight deviation indicate that additional attention is required to consistently improve future performance. Comparing to other countries in the region showed that the Medical City fares much better on integral composites. For instance, Management Support for Patient Safety had a percent positive score of 75.3% while it scored 37% in Palestine [16] and 25.2% in Oman [4]. Moreover, Feedback and Communication about Error received 71.8% percent positive response in Riyadh but scored 46% in Palestine [16]. Some other composites were found to be common areas requiring improvement across the three countries such as Staffing, Communication Openness and Non-Punitive Response to Error.

Of note is the significant association between most safety culture composites and lower number of events report. In fact, only Hospital Management Support for Patient Safety and Hospital Handoffs and Transitions were found to be associated with higher number of events. The significant association between Feedback and Communication about Error and Frequency of events reported is also of note in this context. This indicates that the underlying system that governs these processes may actually improve reporting compared to other patient safety culture composites. Incident and event reporting are critical to maintain patient safety. Hospital staff are often too busy to report, unsure about the mechanisms of reporting or simply insufficiently engaged in the importance of reporting [24].

Another interesting observation is the impact of higher scores hospital management on improved patient safety grade and higher number of events reported. This highlights the importance of managerial commitment particularly as evidence shows a link between administrative support and performance in process of care, lower mortality rates (Jiang et al. 2009), and better overall hospital performance [25–27].

Furthermore, results indicated that pharmacists were almost three times as likely to report events. This is in line with findings in the literature that indicate that pharmacists’ role in in error reporting [28]. Still, this indicates the need to work on improving the reporting process through addressing communication and feedback channels to ensure that pharmacists continue to report [28] and that other staff members are equally inclined to report errors.

To our knowledge, this is the first study to conduct a re-assessment of patient safety culture in Riyadh. Results can provide valuable insight to hospital leaders on how their quality improvement plans over a span of 3 years have affected patient safety culture. Despite using a pre-validated survey which was also provided in Arabic, the values of Cronbach’s Alpha are still considered low and did not improve much compared to the previous assessment [13]. However, it should be noted that they are comparable to a similar assessment in the region where the values were attributed to the use of two languages and the wide range of respondents [14]. Evidence also shows that lower Cronbach’s Alpha values are typically expected with psychological constructs where diverse items are being measured [29].

## Conclusion

Study findings indicate that while tangible improvements were observed, there are still areas that the hospital can enhance in effort to improve overall patient safety culture. Study findings will guide and inform overall strategies to further improve patient safety practices. There is a need to invest further in determinants of patient safety culture, particularly areas that impact event reporting. Results confirm that regular assessment can allow hospitals to better understand how overall performance improved and if any other areas need further enhancement.

## Abbreviations

CI: Confidence interval
HSOPSC: Hospital Survey on Patient Safety Culture
OR: Odds ratio
PS: Patient safety
PSC: Patient safety culture
SD: Standard deviation
US: United States
